# Online procedure to encourage reading in children with school difficulties and their guardians, during the COVID-19 pandemic

**DOI:** 10.1590/2317-1782/20232022056en

**Published:** 2023-12-18

**Authors:** Giulia Ito Silva, Gabriela Diniz, Patrícia Abreu Pinheiro Crenitte, Aline Roberta Aceituno da Costa

**Affiliations:** 1 Departamento de Fonoaudiologia, Faculdade de Odontologia de Bauru, Universidade de São Paulo - USP - Bauru (SP), Brasil.

**Keywords:** Motivation, Reading, Technology, Schools, Pandemics, Child

## Abstract

School is the main environment for the formal promotion of literacy and it is the first step to the world of reading. However, with the occurrence of the COVID-19 pandemic, pedagogical activities had to be suspended or adapted to the remote model, causing harm to all students, and notably to those who already had school difficulties, especially those related to reading. Proficiency and the habit of reading are important conditions for academic development. Studies have shown that the application of strategies in the family context and the use of digital platforms, with the purpose of sharing readings, are practices that promote the reading habit. Therefore, the present study aimed to report the experiences of applying an online procedure to encourage reading in children with school difficulties and their respective guardians (six dyads), using a specific digital platform for sharing readings. The results obtained include a behavioral change in the children towards solid reading habits, a change in mothers' daily attitudes in the family environment towards encouraging reading, and mothers’ encouraging behaviors when sharing their children’s reading. The experiences reported were promising and, to further consolidate the findings, new studies are suggested with a sample size that allows statistical analyses, longitudinal studies and also with a possible face-to-face or hybrid application of the procedure.

## INTRODUCTION

Literacy and initial reading instruction are formally developed in a school environment, which is designed and prepared for this, especially with regard to the initial grades. Therefore, the suspension or adaptation of face-to-face classes to the remote model, due to the COVID-19 pandemic, caused losses for all students, being even more pronounced for those who already had school difficulties^(1)^.

Still as a consequence of the health crisis, in-person services of several health services considered non-essential were interrupted. Users who carried out their rehabilitation in speech therapy outpatient clinics were temporarily no longer assisted due to the need for social distancing^(2)^. Among them, children and adolescents with school difficulties and Learning Disorders.

One of the measures to reverse this situation and encourage the expansion of repertoires would be to promote the reading habit. A study carried out in 2017^(3)^ demonstrated the importance of the family in this process. Parents who valued and committed to promoting reading skills, motivation and habits applied favorable strategies for reader development^(3)^. These strategies included explicit communication about the value of reading, active listening and questioning, the creation of an environment favorable to the habit of reading, the promotion of reader independence and the inclusion of reading practices in the routine^(3)^.

Another study carried out in Pakistan in 2018^(4)^, also brought strategies to encourage reading in the family environment: exposure to books, trips to libraries, developing school subjects at home, reserving moments to practice reading, creating a reading spot, reading aloud and sharing readings^(4)^.

In general, reading is a solitary act and sharing content read does not always occur. Adding possibilities to socialize thoughts and reflections arising from this activity to the reading activity can increase the chance that there will be motivation to carry out new readings and that the content will be remembered in a meaningful way. The literature on the benefits of shared reading is vast and covers the topic from different points of view, with scientific evidence of its benefits for the development of important skills to establish and maintain the reading habit, such as promoting reading comprehension, memory, attention and motivation for the reading activity^(5-7)^.

With technological and new digital platforms progress, the possibilities for sharing stories read are not restricted to informal conversations or reading circles, there are also online book clubs^(8)^, thematic social networks and literary blogs^(9)^.

A study carried out in 2018^(8)^ analyzed discussions about readings that took place outside school facilities, among teenagers aged 13 to 18, in an online book club. The results showed that engagement with the digital platform was positive, as students were involved in the discussions. Furthermore, the tool showed strong potential to become a pedagogical strategy to promote reading and a promising ally for initial reading instruction. This was evidenced by the possibility of self-selection of literature by students, an important variable that guaranteed involvement in discussions and non-simultaneous interactions, made possible by the platform, which allowed participants more time to read and respond to interactions^(8)^.

Therefore, the present study aimed to report the experiences of the online application of a procedure to encourage reading in children with school difficulties and their respective guardians.

## CASE STUDY

Seven patients from a school clinic, aged between six and fourteen years old (Group of patients) and their fathers, mothers and/or guardians were invited to participate. The study was approved by the Human Research Ethics Committee, C.A.A.E. 30007620.8.0000.5417. The volunteers signed the Free and Informed Consent Form (guardians) and the Assent Form (Patients).

The inclusion criteria for participating in the Group of Patients were having access to a mobile device and good quality broadband internet, in addition to having an adult guardian available to attend the virtual meetings. The inclusion criterion for participating in the Group of Adults was being responsible for one of the participants in the Group of Patients.

Six patients (P1, P2, P3, P4, P5 and P6) and their respective mothers agreed to participate in the study. However, as P5 and P6 were siblings, the sample of mothers consisted of five participants (M1, M2, M3, M4 and M5). Participant information (Patients and Mothers) are shown in Table 1.

**Table 1:** Patient profile*

Profile	Patient 1 (P1)	Patient 2 (P2)	Patient 3 (P3)	Patient 4 (P4)	Patient 5 (P5)	Patient 6 (P6)
Age/Grade	12/ 6^th^	12/ 6^th^	13/ 6^th^	6/ Preschool	12/ 7^th^	10/ 5^th^
Gender	Male	Male	Male	Male	Male	Male
Type of School	Public	Private	Private	Private	Public	Public
Literacy	Non-literate	Literate	Literate	Non-literate	Literate	Literate
Diagnosis in Reading and Writing	Specific Learning Disorder	Specific Learning Disorder	Attention Deficit/Hyperactivity Disorder	---	Specific Learning Disorder	Specific Learning Disorder
Time of intervention	2 years	1 year and 6 months	2 years	3 years	1 year and 6 months	2 years
Do you like to read?	More or less	More or less	Nothing	Not so much	Not so much	More or less
What do you read for?	Leisure	Information	Leisure	Leisure	Information	School
Do you ask to buy books?	Never	Sometimes	Never	Sometimes	Never	Never
Do you read materials other than what the school requests?	No	Yes	No	Yes	Yes	Sometimes
Are you used to reading outside of school?	Not regularly	Not regularly	Never	Every day	Never	Not regularly
What do you feel When you read?	That it’s tiring/boring	That it’s fun/interesting	That it’s tiring/boring	That it’s fun/interesting	That it’s tiring/boring	That it’s fun/interesting
Guardian	Mother (M1)	Mother (M2)	Mother (M3)	Mother (M4)	Mother (M5)	Mother (M5)
Mother’s age	44 years old	33 years old	63 years old	36 years old	37 years old	-
Mother’s Education	Elementary - unfinished	High school - finished	Elementary - finished	High school - finished	High school - finished	-
Mother’s profession	Assistant/ cleaning	Manicure	Administrative	Housewife	Self-employed	-

* The reader profile questions were taken from the work of Zorzi et al.^(10)^

P1, P2, P5 and P6 had previously been diagnosed with Specific Learning Disorder and P3 was diagnosed with Attention-Deficit/Hyperactivity Disorder. The diagnoses were made by a specialized team from the school clinic and they had 18 to 24 months of speech therapy intervention.

P4 was referred to the teaching clinic for preventive intervention. Due to the pandemic, the child, of Chinese descent and whose first language (L1) is Mandarin, spent seven months deprived of the Portuguese language (L2) with losses in pragmatics, syntax, semantics, phonology and vocabulary in L2. If the intervention were not carried out, the child would not be able to advance in school in 2021 to the first year of elementary school, as they attend a Brazilian school in which L2 is their only option.

The intervention procedure with the Group of Patients and the Group of Mothers occurred concomitantly, and some activities occurred together. All stages took place remotely, using a specific free digital platform to encourage the sharing of readings asynchronously (clube-da-leitura.web.app) and the Google Meet platform for synchronous meetings.

Stages carried out with the Group of Patients and with the Group of Mothers:

Stage 1: Patients were asked to answer the “Reading Profile Questionnaire”^(10)^ (shown in Table 1) and the “Reading Behavior Scale” (shown in Table 2), which was prepared by the researchers and covers questions on social behaviors based on notes found in the literature^(4,5)^ about promoting reading habits.

**Table 2:** Responses obtained on the Reading Behavior Scale

**Patient**	**Moment of application**	**Reading Behavior Scale**				
		Question 1	Question 2	Question 3	Question 4	Question 5
		What are the chances of you asking for a book as a birthday, Christmas or Children's Day gift?	When you're out for a walk with your parents or a family member, what's the chance that you'll ask to go into a bookstore or library to look at some books?	After reading a book, magazine, comic book or text, what are the chances of you telling your parents about what you read?	After reading a book, magazine, comic book or text, what are the chances of you telling your friends about what you read?	In the in-person classroom, if your teacher asks “Who would like to read to the class?”, what are the chances of you raising your hand and reading?
P1	Pre	3	4	2	1	2
P1	Pos	3	2	3	2	1
P2	Pre	2	3	4	4	2
P2	Pos	3	5	5	4	2
P3	Pre	1	1	2	5	5
P3	Pos	2	2	4	5	5
P4	Pre	4	4	4	2	3
P4	Pos	3	3	4	2	3
P5	Pre	2	2	2	1	1
P5	Pos	3	2	2	2	2
P6	Pre	3	1	1	1	3
P6	Pos	2	1	1	2	3

Caption: The moment of application of the Scale was before and after the reading incentive procedure. The standardization of scale responses was: 1 - “None”, 2 - “Little”, 3 - “Maybe there is a chance”, 4 - “Big” and 5 - “Very big”

Then, patients chose a virtual book from the titles available on free websites^(11-14)^.

The book was read, in the case of literate children, or narrated, in the case of non-literate children, to the mother. The session was recorded and analyzed by researchers who filled out the “Check-list - Parents’ behaviors regarding their child’s reading report”. This instrument consists of the following questions: “Question 1 - Does the family member make eye contact with the child?”, “Question 2 - Does the family member ask pertinent questions?”, “Question 3 - Does the family member encourage the child’s response?”, “Question 4 - Does the family member smile at the child?”, “Question 5 - Does the family member nod?”, “Question 6 - Does the family member present other behaviors in response to the report?”.

The objective of reading the virtual book, in this activity, was to encourage the child to share a story they read and record the guardian's behavior in these situations.

Thus, Stage 1 of the Group of Mothers was carried out through participation in an individual session with their respective children. The objective was to observe the mother's behavior during their child's reading.

### Results of stage 1

All dyads completed the stage in two sessions of 30 minutes each. The answers to the “Reader Profile Questionnaire”^(9)^ and personal data are summarized in Table 1. For P1 and P4, who were not literate, the books available had no texts. The children interpreted the images and told the story to their mothers. P2, P3, P5 and P6 read the chosen books. The analysis of the recordings of interactions between mother-child dyads is presented with the results of Stage 1 of the Group of Mothers.

Furthermore, the patients wrote a personal presentation, which was read by one of the researchers, a speech therapist, who accompanied all participants throughout all the activities and who will from now on be referred to as Mediator. After the reading, the Mediator made comments valuing the productions and models on how to promote cohesion, coherence, punctuation and spelling of the text. This text was made available on the asynchronous platform, with a photograph, so that participants could get to know each other. P1 and P4, as they did not have autonomy in writing, expressed verbally what they would like to convey and the message was written by the Mediator.

The analysis of the six videos of the dyads showed that none of the mothers made eye contact with their child during the reading, nor did they ask pertinent questions about it. Only M3 encouraged the response. M1, M3 and M4 smiled at their children. As for the act of nodding, only M4 did it. Finally, other behaviors were visualized, such as: showing interest in the activity (M1, M2, M3, M4 and M5), making additional comments (M1 and M2) and helping with the reading (M3).

Stage 2: Each child was invited to choose another child to interact with in future stages and then they were asked to choose and read a virtual book.

Stage 2 of the Group of Mothers consisted of a conversation circle in which all participants were invited to introduce themselves, explain experiences or doubts and were guided on the activities that were being carried out with their children. At this same meeting, a digital folder was made available containing the six topics: Importance of being a model reader, Strategies for reading together, Suggestions of actions to include reading in the routine, Sharing of readings by the family, Self-selection of the book and Tips to encourage the sharing of readings (pointed out in the literature as useful^(3,4)^.

They were then invited to respond to the “Questionnaire to verify daily attitudes to encourage reading in the family environment” made available virtually via the Google Forms tool. In the presentation of the digital folder, each topic was explained and exemplified.

Finally, four websites with free virtual books were presented so that they could choose a book and read the story with their respective child, applying the strategies discussed at the meeting.

### Results of stage 2

The choice, made by the patients, of another participant to interact with in future stages was made by observing the photographs and reading the presentation sentences previously prepared and posted in Stage 1. In the case of P1 and P4, the reading of the sentence was carried out by the Mediator.

The reading of the books took a 30-minute session. P2, P3, P5 and P6 read their books orally to the Mediator. P1 chose a book with written text. Therefore, the book was read by the Mediator with the help of the patient. P4 chose a book without text and the narration was carried out by them with occasional interventions from the Mediator.

The Group of Mothers’ meeting lasted 30 minutes and counted on the participation and engagement of all the mothers, who introduced themselves and spoke about their children's difficulties and what they expected from the meeting. They confirmed their satisfaction in being part of the procedure and their desire to know how to promote their children's reading habits. After explaining the digital folder, questions were asked and everyone reported what they were already doing and what they would like to practice on the topics covered.

The “Questionnaire to check daily attitudes to encourage reading in the family environment” consisted of three questions and was answered by M1, M2, M3 and M4. The first question was “How often do you share the material you have read with your child?” M1 and M4 answered “always” and M2 and M3 “rarely”. The second question was “Does your child share readings that they have read or learned?”, to which the answers were “yes” by M2 and M4, and “sometimes” by M1 and M3. Finally, the third question was “What are your behaviors regarding the reading information brought by your child?” and provided five options that could be selected by the interviewee, namely: I stop what I am doing to listen to my child; I listen to my child while carrying out another activity; I add more information; I ask further questions; and I give compliments. There could be more than one answer to this question. M2 stops what she is doing to listen to her child; M1, M3 and M4 listen to their children while carrying out another activity; M4 adds more information and asks further questions; M3 and M4 praise their children.

Stage 3: In stage three, each patient was invited to prepare a written summary about the book read in Step 2 and send it through the asynchronous platform to the patient who chose them.

In this stage, the Group of Mothers participated in a conversation circle in which they were encouraged to share experiences regarding the book read and to use the strategies discussed in the previous meeting. Inthe end, they were asked to answer again the “Questionnaire to verify daily attitudes to encourage reading in the family environment”.

### Results of stage 3

Patient group: It took two sessions of 30 minutes each for patients to complete the summaries and send them to the patients who chose them.

With P1 and P4, retelling strategies were carried out using pictures, so that they felt encouraged to narrate the stories to the Mediator and she could transcribe them in a summary format. With the other patients, the Mediator carried out oral reading comprehension activities to ensure that the story had been understood. In the same session, each patient wrote a summary of their book on a sheet of paper, photographed it and sent it to the Mediator. In the following session, the Mediator valued textual production, worked on improvement aspects and made spelling adjustments to the standard based on reflections with the child, when necessary.

Group of Mothers: the time interval between meetings two and three was seven days. M2, M3 and M4 participated in this meeting, which lasted 20 minutes. Only M4 read together with her child, as proposed in meeting two. She reported that her child chose the book and that this was a factor that motivated them to read. She added that the fact that the book was virtual increased their interest. Among the six topics covered in meeting two, the mother looked for: joint reading strategies, self-selection of the book and tips for encouraging shared reading.

A relevant aspect mentioned by the mother was that the seven-day interval was positive, as it motivated her to apply what she had learned in the previous meeting in a short period of time. The biggest challenge is balancing household chores with educating and stimulating children.

M2 pointed out that, despite not having carried out the reading activity with her child, she applied some strategies discussed in the group of mothers during the seven-day break and encouraged her eldest daughter, who has a reading habit, to talk more about reading with her brother (P2) and read together. She stated that the whole family already has the habit of talking about reading, but now they stop what they are doing to listen to reports of their children's reading.

M3 stated that she was a model reader, but never noticed that she did not share her child's reading. She further added that she was motivated to practice the guidelines provided in meeting two, especially reading together. She also highlighted the importance of the group of mothers, as she believed that just giving her son a book would be enough to make him read. However, at that moment, she understood the complex dimensions involved in the habit of reading and stated that she was committed to practicing strategies to encourage the sharing of readings.

Only M3 and M4 answered the “Questionnaire to verify daily attitudes to encourage reading in the family environment”. When comparing M3's answers to the questionnaire in meeting one (pre-intervention) and meeting three (post-intervention), it was noted that, in question one, “How often do you usually share the material you read with your child?”, the answer was changed from “rarely” to “always”. M4 kept the same answers at both moments. However, in question three, “What are your behaviors regarding the reading information brought by your child?”, a new behavior was checked, namely “I stop what I am doing to listen to my child”.

Stage 4: Finally, in stage four, each participant was asked to read the summary received, record a thank-you video with comments on the story and send it to their colleague via the asynchronous platform. The mother was also asked to retell the book read in stage 2 and fill out the “Reading Behavior Scale”. The retelling was recorded and analyzed by researchers who filled out the “Check-list - Parents’ behaviors regarding their child’s reading report”. Therefore, in this Stage, the Group of Mothers participated as listeners to the story of the book read by their child in Stage 2.

### Results of stage 4

This stage took place in two 30-minute sessions. In the first session, the summary was read, recorded and sent to the colleague, and in the second the patients watched the video sent by the colleague, retold the book read in Stage 2 to the mothers and completed the Reading Behavior Scale.

To understand the summary received, non-literate patients needed the Mediator's help to read it, and literate patients read it orally. Regarding the videos they received, patients stated, in different ways, that they were excited to meet their colleagues through the recorded videos and to hear them about the book they read. The result obtained with the “Reading Behavior Scale”, pre- and post-application of the procedure, can be seen in Table 2.

Patients P2, P4, P5 and P6 had the presence of their respective mothers in the session to retell the second virtual book read. The interactions of the four dyads (M3-P3, M4-P4, M5-P5, M5-P6) were analyzed. There was eye contact, pertinent questions and smiles on the part of the mothers in the four dyads; and encouragement to respond and nod in three dyads (M3-C3, M4-C4, M5-C6), which is different from the records in Stage 1 in which no eye contact was observed and there was only one record of encouragement to respond and nod. Regarding the presence of other behaviors, there was a demonstration of interest and involvement (M3-C3), hugs and participation from the mother throughout the reading (M4-C4).

## DISCUSSION

The prompt agreement to participate in the reading intervention activities by mothers and patients, the engagement of all invited dyads and the non-withdrawal can be indicators that the procedure, the conduct of the topics and the activities were of interest and appropriate for the purpose presented. They also suggest that reading behavior is not necessarily aversive, but presupposes procedures that favor engagement, unlike activities related to oral communication, which in itself is natural to human communication.

In this sense, it is important to highlight that the procedure did not prove to be aversive even for children with school problems related to literacy and who face difficulties in dimensions other than those imposed by social distancing^(15)^. Dealing with the reading situation for these students means facing difficulties with phonological working memory, access to the lexicon in long-term memory and metaphonological skills, to name the main ones^(16,17)^.

Taking into account the pandemic situation, the fully online format was a positive choice for patients to continue to be monitored, as it allowed stimulation in written language as well as the reading habits, thus continuing the activities according to all previous assessments and interventions.

Another advantage of this format, which may have contributed to non-withdrawal and engagement, was greater flexibility in schedules and reduced costs for families, such as travel costs. These aspects were also observed in a Brazilian study^(2)^.

However, for the online application to be possible, it was necessary to apply strict inclusion criteria, meaning that few patients were eligible. An important discussion about access to the internet and technologies is that it is not yet a reality for all socioeconomically disadvantaged patients^(15)^, being an important limitation.

Another factor that led to the exclusion of potential participants was the lack of availability of an adult to accompany the participant during remote sessions. Despite knowing the impact of excluding potential participants from the study, the researchers maintained the decision to control this variable. The importance and impact of monitoring activities carried out at home have been discussed and highlighted as a determining success criteria regarding both the virtual^(18)^ and in-person contexts, as well as regarding children with and without school difficulties.

The cases reported concern children and adolescents with a history of difficulties in the process of learning written language, which makes monitoring even more relevant, since some written commands are not possible and also because the adult's participation not only facilitates the intervention itself, but also allows this adult to start acting in a way connected with the therapeutic proposals in many other everyday situations. The results of this study support the idea that engagement may have been favored by the active participation of the adults.

The proposal was for the child to experience reading three books chosen by themselves. The possibility of self-selection of literature being an important factor in ensuring engagement^(8)^ was observed throughout the sessions and in the report of one of the participating mothers: “The book my child chose was: *Um Medo para Arrepiar*, about monsters, which in itself aroused his interest.” Therefore, this factor is considered important for both clinical and pedagogical practice, as well as a stimulus in a family environment.

Another aspect of the online platform used, which is in line with a study already presented^(8)^, is the use of the asynchronous modality in interactions between users. Non-simultaneous interactions allowed more time for reading and more care when writing^(8)^. The asynchronicity was positive, as it allowed the Mediator to work on the particularities of each child's reading and writing practice.

Furthermore, the implementation of audiovisual resources in interactions in virtual book clubs to assist those who have difficulties with written language^(8)^ was used and facilitated everyone's participation and proximity between participants who experienced a completely virtual interaction there.

A relevant consideration concerns the role played by the Mediator. When analyzing the results, it is possible to observe that the Mediator conducted the proposed activities and made adaptations according to the needs and particularities of each child's reading and writing. The same situation occurred in the context of conversation circles with mothers, where sensitivity was necessary to earn participants’ trust and make them feel comfortable sharing their experiences and daily difficulties, in addition to ensuring equal participation for all. The Mediator ensured that the sharing of experiences was prioritized in a collective construction of learning, which proved to be necessary even in the context of mothers.

Analyzes of mothers' responses in conversation circles show that they understand that family support is essential for the development of the reading habit. However, initially, they presented few of the behaviors identified as facilitators in the literature and, after some meetings on the topic, they began to incorporate important actions in their interaction with their child. The present study took into account the notes from previous studies to plan the topics to be taken to the companions^(4,5)^. When addressing issues related to the habit of reading, some aspects become fundamental, being addressed in meeting two of the Group of Mothers' methodology: explicit communication about the value of reading, active listening and questioning, the creation of an environment favorable to the habit of reading, the incorporation of reading practices into the routine^(3)^, the importance of exposure to books, addressing school subjects at home, setting aside time to practice reading, practicing reading aloud and sharing readings^(4)^. In this way, a discussion arises about the importance of not only having a companion for home activities, but also the need to expand the repertoire of family members, so that this support can be better utilized. The results of this study show that when there is a joint construction of a repertoire of daily attitudes that facilitate learning, there is greater engagement on the part of the family member, thus increasing the chance that collaboration with the children's activities will occur in a less aversive way for everyone.

When comparing the pre- and post-intervention reading behavior scale, a positive change is noticed in the response to at least two situations for five children. In other words, after the procedure, five of them started to respond positively to behaviors that make them more likely to develop reading behaviors. It is important to highlight that it is possible that such a change occurred, at first, only in the scope of verbal behavior, but no changes have yet occurred in routine actions. In this sense, it would be important that future follow-ups were carried out with the dyad.

Another analysis is the change in behavior of the four mothers who participated in meetings one and four of the methodology. This change concerns the presence of behaviors in meeting four that were not demonstrated in meeting one, such as: eye contact, questioning, encouraging answers, smiling and nodding. Among the four mothers, all had participated in meeting two and only two in meeting three. This fact generates reflection on the importance of meeting two for changing these mothers’ attitudes and the short and long-term consequences for these children's reading habits.

Still regarding the group of mothers, it is important to highlight the report of the change in daily attitudes to encourage reading pointed out by the two mothers who responded to the questionnaire. One mother started to “always” share her reading with her child and the other started to “stop what she is doing to listen to her child”, as these are important changes to promote the reading habit.

It is suggested that similar studies be carried out with a sample size that allows statistical analyzes in order to map the effects of the procedure, longitudinal studies with exposure of participants to more books in order to verify the presence of changes in the scales of reading behaviors after the stimulation time and the application of this procedure in a face-to-face model.

Finally, a final reflection brings us to the fact that the pandemic has imposed innovations in several areas and in the area of Speech Therapy with regard to written language it was no different. Although the objective of this study was specifically to report the experiences of the online application of a procedure to encourage reading in children with school difficulties and their respective guardians, the results of the study add to others^(19)^ that reinforce the possibility of carrying out fruitful interventions through tele-speech therapy, an important perspective from an economic, social and educational point of view and which deserves special attention from this historical moment that the entire area of health and education is going through.

## FINAL COMMENTS

The learning of children and adolescents with school difficulties was even more compromised during the COVID-19 pandemic period. One way to reduce the damage caused to written language is through reading.

This report showed the benefits of a speech therapy procedure applied in an online format with a focus on sharing readings through a digital platform and encouraging reading in the family environment. The results obtained include the change in behavior of the children involved towards a greater propensity for reading habits, the report of changes in mothers’ daily attitudes in the family environment towards encouraging reading, and the presence of encouraging behaviors on the part of mothers when it comes to sharing their children’s readings.

The experiences reported were promising. To further consolidate the findings, new studies are suggested with a sample size that allows statistical analysis, longitudinal studies that measure the long-term impact of the procedure and also face-to-face or hybrid application of the procedure.

## References

[1] Barros FC, Vieira DAP (2021). Os desafios da educação no período de pandemia. Braz J Dev..

[2] Dimer NA, Canto-Soares N, Santos-Teixeira L, Goulart BNG (2020). Pandemia do COVID-19 e implementação de telefonoaudiologia para pacientes em domicílio: relato de experiência. CoDAS.

[3] Capotosto L, Kim JS, Burkhauser MA, Oh Park S, Mulimbi B, Donaldson M (2017). Family support of third-grade reading skills, motivation, and habits. AERA Open.

[4] Bano J, Jabeen Z, Qutoshi SB (2018). Perceptions of teachers about the role of parents in developing reading habits of children to improve their academic performance in schools. J Educ Educ Dev..

[5] Menotti ARS, Domeniconi C, Costa ARA (2019). Capacitação de professores do ensino infantil para o uso de estratégias bem-sucedidas de leitura compartilhada. CoDAS.

[6] Towson J, Fettig A, Fleury VP, Abarca DL (2017). Dialogic reading in early childhood settings: a summary of the evidence base. Top Early Child Spec Educ.

[7] Kim SY, Rispoli M, Lory C, Gregori E, Brodhead MT (2018). The effects of a shared reading intervention on narrative story comprehension and task engagement of students with autism spectrum disorder. J Autism Dev Disord.

[8] Colwell J, Woodward L, Hutchison A (2018). Out-of-school reading and literature discussion: an exploration of adolescents’ participation in digital book clubs online. Learning..

[9] García-Roca A (2020). Spanish reading influencers in goodreads: participation, experience and canon proposed. J New Appr Educ Res..

[10] Zorzi JL, Serapompa MT, Faria AT, Oliveira PS (2004). A influência do perfil de leitor nas habilidades ortográficas. Psicopedagogia.

[11] Itaú (2022). Leia para uma criança.

[12] O Pequeno Leitor (2022). http://www.opequenoleitor.com.br.

[13] Escola Games (2022). http://www.escolagames.com.br/livros.

[14] Laboratório de Educação (2022). Espaço de leitura.

[15] Marcon K (2020). Inclusão e exclusão digital em contextos de pandemia: que educação estamos praticando e para quem?. Criar Educação.

[16] Santos IMS, Roazzi A, Melo MRA (2020). Consciência fonológica e funções executivas: associações com escolaridade e idade. Psicol Esc Educ.

[17] Silva C, Capellini SA (2021). Correlação de habilidades cognitivo-linguísticas de escolares submetidos a intervenção fonológica. Psicopedagogia.

[18] Von Stein D, Bianchini LGB, Mazzafera BL, Yaegashi SFR, Oliveira LV (2020). Experiências de uma professora: atividades remotas com alunos da sala de recursos multifuncionais durante a pandemia do coronavírus. Práxis.

[19] Dimer NA, Canto-Soares N, Santos-Teixeira L, Goulart BNG (2020). Pandemia do COVID-19 e implementação de telefonoaudiologia para pacientes em domicílio: relato de experiência. CoDAS.

