# Supplementary material for: Online procedure to encourage reading in children with school difficulties and their guardians, during the COVID-19 pandemic
Source: Codas. 2023 Dec 18;36(1):e20220056. doi: 10.1590/2317-1782/20232022056en (PMC10750858; doi:10.1590/2317-1782/20232022056en)
Supplement: Translation

# Translation: Procedimento *online* de incentivo à leitura em crianças com dificuldades escolares e seus responsáveis, durante a pandemia de COVID-19

**DOI:** 10.1590/2317-1782/20232022056pt

**Authors:** Giulia Ito Silva, Gabriela Diniz, Patrícia Abreu Pinheiro Crenitte, Aline Roberta Aceituno da Costa

**Affiliations:** 1 Departamento de Fonoaudiologia, Faculdade de Odontologia de Bauru, Universidade de São Paulo - USP - Bauru (SP), Brasil.

**Keywords:** Motivação, Leitura, Tecnologia, Instituições Acadêmicas, Pandemias, Criança

## Abstract

A escola é o principal ambiente para a promoção formal da alfabetização e do letramento, os quais são portas para o mundo da leitura. Porém, com a ocorrência da pandemia do COVID-19, as atividades pedagógicas tiveram que ser suspensas ou adaptadas ao modelo remoto, acarretando prejuízos a todos os alunos, e principalmente àqueles que já apresentavam dificuldades escolares, especialmente as relacionadas à leitura. Sabe-se que a proficiência e o hábito de leitura são condições importantes para o desenvolvimento acadêmico geral. Estudos têm demonstrado que a aplicação de estratégias no contexto familiar e a utilização de plataformas digitais, com a finalidade de compartilhamento de leituras, são práticas favorecedoras do hábito leitor. Dessa forma, o presente estudo objetivou relatar as experiências da aplicação *online* de um procedimento de incentivo à leitura, em crianças com dificuldades escolares e seus respectivos responsáveis (seis díades), com a utilização de uma plataforma digital específica para compartilhamentos de leituras. Dentre os resultados obtidos estão: a mudança de comportamentos das crianças envolvidas para maior propensão a hábitos leitores, a mudança de atitudes diárias das mães no ambiente familiar para o incentivo à leitura, e a presença de comportamentos incentivadores por parte das mães, frente ao compartilhamento de leituras de seus filhos. As experiências relatadas mostraram-se promissoras e para maior consolidação dos achados, sugerem-se novos estudos com número amostral que permita análises estatísticas, estudos longitudinais e também com uma possível aplicação presencial ou híbrida do procedimento.

## INTRODUÇÃO

A alfabetização e o letramento são desenvolvidos, formalmente, em ambiente escolar, o qual é pensado e preparado para tanto, especialmente no que diz respeito às séries iniciais. Por isso, a suspensão ou adaptação das aulas presenciais ao modelo remoto, devido à pandemia do COVID-19, acarretou prejuízos a todos os alunos, sendo ainda mais acentuada para aqueles que já apresentavam dificuldades escolares^(1)^.

Ainda como consequência da crise sanitária, houve a interrupção de atendimentos presenciais de vários serviços de saúde considerados não essenciais. Usuários que realizavam suas reabilitações em ambulatórios de fonoaudiologia deixaram de ser assistidos, temporariamente, diante da necessidade de distanciamento social^(2)^. Dentre eles, crianças e adolescentes com dificuldades escolares e Transtornos de Aprendizagem.

Uma das medidas para reverter esse panorama e favorecer a ampliação de repertórios, seria promover o hábito leitor. Um estudo realizado em 2017^(3)^ demonstrou a importância da família neste processo. Os pais que valorizavam e se empenhavam na promoção de habilidades, motivação e hábitos de leitura aplicavam estratégias favoráveis para o desenvolvimento do leitor^(3)^. Dentre essas estavam: a comunicação explícita sobre o valor da leitura, a realização de escuta ativa e questionamentos, a criação de um ambiente favorável ao hábito de ler, a promoção da independência do leitor e a incorporação de práticas de leitura na rotina^(3)^.

Outro estudo, realizado no Paquistão em 2018^(4)^, também trouxe estratégias para o incentivo da leitura no ambiente familiar: exposição a livros, passeios a bibliotecas, desenvolvimento de assuntos escolares em casa, reserva de momentos para prática da leitura, criação de um espaço para leitura, realização de leitura em voz alta e compartilhamento de leituras^(4)^.

Em geral, a leitura é um ato solitário e o compartilhamento de conteúdos lidos nem sempre ocorre. Agregar, à atividade de leitura, possibilidades de socializar pensamentos e reflexões advindas desta, pode aumentar a chance de que haja motivação para a realização de novas leituras e de que o conteúdo seja relembrado de forma significativa. A literatura sobre os benefícios da leitura compartilhada é vasta e abarca o tema de diversos pontos de vista, com evidências científicas de seus ganhos para o desenvolvimento de habilidades importantes para instalar e manter o hábito leitor, como favorecer a compreensão leitora, a memória, a atenção e a motivação para a atividade da leitura^(5-7)^.

Com o avanço tecnológico e de novas plataformas digitais, as possibilidades de compartilhamento de histórias lidas não se restringem às conversas informais ou às rodas de leitura, há também os clubes do livro *online* ^(8)^, redes sociais temáticas e blogs literários^(9)^.

Um estudo realizado em 2018^(8)^ se propôs a analisar as discussões sobre leituras realizadas fora da escola, entre adolescentes de 13 a 18 anos, em um clube do livro *online*. Os resultados demonstraram que o envolvimento com a plataforma digital foi positivo, uma vez que os estudantes se mostraram engajados com as discussões. Além disso, a ferramenta se mostrou com forte potencial para se tornar uma estratégia pedagógica de promoção da leitura e uma promissora situação de letramento. O que ficou evidenciado pela possibilidade de autosseleção da literatura pelos estudantes, variável importante que garantia o envolvimento nas discussões e as interações não-simultâneas, possibilitadas pela plataforma, que permitiam que os participantes tivessem mais tempo para ler e responder às interações^(8)^.

Diante destas considerações, o presente estudo objetivou relatar as experiências da aplicação *online* de um procedimento de incentivo à leitura, em crianças com dificuldades escolares e seus respectivos responsáveis.

## APRESENTAÇÃO DO CASO

Foram convidados a participar, sete pacientes de uma Clínica escola, na faixa etária de seis a catorze anos (Grupo de pacientes) e seus pais, mães e/ou responsáveis. O estudo foi aprovado pelo Comitê de Ética em Pesquisa com Seres Humanos, C.A.A.E. 30007620.8.0000.5417. Os voluntários assinaram o Termo de Consentimento Livre e Esclarecido (responsáveis) e o Termo de assentimento (Pacientes).

O critério de inclusão para compor o Grupo de pacientes era possuir acesso a um dispositivo móvel e à internet de banda larga de boa qualidade, além de haver um responsável maior de idade com disponibilidade para acompanhar os encontros virtuais. O critério de inclusão para participar do Grupo adulto, era ser responsável por um dos participantes do Grupo de pacientes.

Aceitaram participar do estudo seis pacientes (P1, P2, P3, P4, P5 e P6) e suas respectivas mães. No entanto, como P5 e P6 eram irmãos, a amostra de mães foi composta por cinco participantes (M1, M2, M3, M4, M5). As informações dos participantes (Pacientes e Mães) encontram-se na Tabela 1.

**Tabela 1:** Perfil dos pacientes*

Perfil	Paciente 1 (P1)	Paciente 2 (P2)	Paciente 3 (P3)	Paciente 4 (P4)	Paciente 5 (P5)	Paciente 6 (P6)
Idade/ Série	12/ 6°	12/ 6°	13/ 6°	6/ Ensino Infantil	12/ 7°	10/ 5°
Sexo	Masculino	Masculino	Masculino	Masculino	Masculino	Masculino
Tipo Escola	Pública	Particular	Particular	Particular	Pública	Pública
Alfabetização	Não Alfabetizado	Alfabetizado	Alfabetizado	Não Alfabetizado	Alfabetizado	Alfabetizado
Diagnóstico em Leitura e Escrita	Transtorno Específico de Aprendizagem	Transtorno Específico de Aprendizagem	Transtorno do Déficit de Atenção e Hiperatividade	---	Transtorno Específico de Aprendizagem	Transtorno Específico de Aprendizagem
Tempo de intervenção	2 anos	1 ano e 6 meses	2 anos	3 meses	1 ano e 6 meses	2 anos
Gosta de ler?	Mais ou menos	Mais ou menos	Nada	Pouco	Pouco	Mais ou menos
Para que você lê?	Lazer	Informativa	Lazer	Lazer	Informativa	Escolar
Você pede para comprar livros?	Nunca	Às vezes	Nunca	Às vezes	Nunca	Nunca
Você lê outros materiais além do que a escola solicita?	Não	Sim	Não	Sim	Sim	Às vezes
Você está acostumado a ler fora da escola?	Não regularmente	Não regularmente	Nunca	Todos os dias	Nunca	Não regularmente
O que você sente quando lê?	Que é cansativo/chato	Que é gostoso/interessante	Que é cansativo/chato	Que é gostoso/interessante	Que é cansativo/chato	Que é gostoso/interessante
Responsável	Mãe (M1)	Mãe (M2)	Mãe (M3)	Mãe (M4)	Mãe (M5)	Mãe (M5)
Idade da Mãe	44 anos	33 anos	63 anos	36 anos	37 anos	-
Escolaridade da Mãe	Fundamental incompleto	Ensino Médio completo	Fundamental completo	Ensino médio completo	Ensino médio completo	-
Profissão da Mãe	Auxiliar/ limpeza	Manicure	Administrativo	Do lar (sic)	Autônoma	-

* As questões do perfil leitor foram retiradas do trabalho de Zorzi et al.^(10)^

P1, P2, P5 e P6 haviam sido previamente diagnosticados com Transtorno Específico de Aprendizagem e P3 apresentava diagnóstico de Transtorno do *Déficit* de Atenção e Hiperatividade. Os diagnósticos foram realizados por uma equipe especializada da clínica-escola e eles apresentavam de 18 a 24 meses de intervenção fonoaudiológica.

P4 foi encaminhado para a clínica-escola, para a realização de intervenção de caráter preventivo. Devido à pandemia, a criança, que tem descendência chinesa e como primeira língua (L1) o Mandarim, passou sete meses privada da língua portuguesa (L2) com prejuízos em pragmática, sintaxe, semântica, fonologia e vocabulário na L2, e caso não fosse realizada intervenção, não poderia avançar na série escolar no ano de 2021, para o primeiro ano do ensino fundamental já que cursa uma escola brasileira na qual a L2 é sua única opção.

O procedimento de intervenção com o Grupo de pacientes e com o Grupo de mães ocorreu de forma concomitante, algumas atividades ocorreram em conjunto. Todas as etapas ocorreram de forma remota, utilizando-se de uma plataforma digital gratuita específica para incentivo ao compartilhamento de leituras de forma assíncrona (clube-da-leitura.web.app) e da plataforma *Google Meet,* para encontros síncronos.

Etapas realizadas com o Grupo de pacientes e com o Grupo de mães:

Etapa 1: Foi solicitado aos pacientes que respondessem o “Questionário Perfil Leitor”^(10)^ (cujas questões estão apresentadas na Tabela 1) e as questões da “Escala de Comportamentos Leitores” (disponível na Tabela 2) a qual foi elaborada pelos pesquisadores e abarca questões sobre comportamentos sociais, baseados nos apontamentos encontrados na literatura^(4,5)^ sobre promoção do hábito de leitura.

**Tabela 2:** Respostas obtidas na Escala de Comportamentos Leitores

**Paciente**	**Período de Aplicação**	**Escala de comportamentos leitores**				
		Questão 1	Questão 2	Questão 3	Questão 4	Questão 5
		Quando chega o seu Aniversário, o Natal ou o Dia das Crianças, qual a chance de você pedir um livro de presente?	Quando você está passeando com seus pais ou alguém da família, qual a chance de você pedir para entrar em uma livraria ou biblioteca para ver alguns livros?	Depois de ler um livro, uma revista, um gibi ou texto, qual a chance de você contar sobre o que você leu para seus pais?	Depois de ler um livro, uma revista, um gibi ou texto, qual a chance de você contar sobre o que você leu para seus amigos?	Na sala de aula presencial ou, se sua professora perguntar “Quem gostaria de ler para a turma?” qual a chance de você levantar a mão e fazer a leitura?
P1	Pré	3	4	2	1	2
P1	Pós	3	2	3	2	1
P2	Pré	2	3	4	4	2
P2	Pós	3	5	5	4	2
P3	Pré	1	1	2	5	5
P3	Pós	2	2	4	5	5
P4	Pré	4	4	4	2	3
P4	Pós	3	3	4	2	3
P5	Pré	2	2	2	1	1
P5	Pós	3	2	2	2	2
P6	Pré	3	1	1	1	3
P6	Pós	2	1	1	2	3

Legenda: O momento da aplicação da Escala foi pré e pós procedimento de incentivo à leitura. A padronização nas respostas da escala foram: 1 - “Nenhuma”, 2 - “Pouca”, 3 - “Talvez tenha uma chance”, 4 - “Grande” e 5 - “Muito grande”

Em seguida, os pacientes escolheram um livro virtual^(11-14)^ dentre os títulos disponibilizados, em sites gratuitos.

O livro foi lido, no caso dos alfabetizados, ou narrado, no caso dos não alfabetizados, para a mãe. A sessão foi gravada e analisada pelos pesquisadores que preencheram o “*Check-list -* Comportamentos dos pais frente ao relato da leitura do filho”. Este instrumento é composto pelas seguintes questões: “Questão 1 - Familiar faz contato ocular com a criança?”, “Questão 2 - Faz perguntas pertinentes?”, “Questão 3 - Incentiva a resposta da criança?”, “Questão 4 - Sorri para a criança?”, “Questão 5 - Assente com a cabeça?”, “Questão 6 - Apresentou outros comportamentos frente ao relato?”.

O objetivo da leitura do livro virtual, nesta atividade, era incentivar a criança a compartilhar uma história lida por ela e registrar o comportamento do responsável nessas situações.

Assim, a Etapa 1 do Grupo de mães foi realizada por meio da participação, em sessão individual com seus respectivos filhos. O objetivo era observar o comportamento da mãe frente ao ato de ler do filho.

### Resultados da etapa 1

Todas as díades completaram a etapa em duas sessões de 30 minutos cada. As respostas ao “Questionário Perfil Leitor”^(9)^ e os dados pessoais estão resumidos na Tabela 1. Para P1 e P4, não alfabetizados, os livros disponibilizados eram sem textos. Eles interpretaram as imagens e contaram a história para suas mães. Já P2, P3, P5 e P6 leram os livros escolhidos. A análise das gravações das interações entre as díades mãe-filho está apresentada junto aos resultados da Etapa 1 do grupo de mães.

Além disso, os pacientes redigiram uma apresentação pessoal, a qual foi lida por uma das pesquisadoras, fonoaudióloga, que acompanhou todos os participantes ao longo de todas as atividades e que será de agora em diante referida como Mediadora. Após a leitura, a Mediadora apresentou comentários de valorização das produções e modelos de como favorecer a coesão, coerência, pontuação e a ortografia do texto. Esse texto foi disponibilizado na plataforma assíncrona, com uma fotografia, para que os participantes pudessem se conhecer. P1 e P4, por não terem autonomia na escrita, expressaram verbalmente o que gostariam de transmitir e a mensagem foi redigida pela Mediadora.

A análise dos seis vídeos das díades mostrou que nenhuma das mães fez contato ocular com o seu filho durante a leitura, nem realizaram perguntas pertinentes a respeito da mesma. Apenas M3 incentivou a resposta. M1, M3 e M4 sorriram para os seus filhos. Quanto ao ato de assentir com a cabeça, apenas M4 o realizou. Por fim, outros comportamentos foram visualizados, como: demonstração de interesse pela atividade (por M1, M2, M3, M4 e M5), realização de comentários complementares (por M1 e M2) e auxílio na leitura (por M3).

Etapa 2: Cada criança foi convidada a escolher uma outra para interagir em etapas futuras e em seguida foi solicitado que escolhessem e fizessem a leitura de um livro virtual.

A Etapa 2 do Grupo de mães constou de uma dinâmica de roda de conversa em que todas as participantes foram convidadas a se apresentar, expor experiências ou dúvidas e foram orientadas quanto às atividades que estavam sendo realizadas com os filhos. Neste mesmo encontro, foi disponibilizado um folder digital que continha os seis tópicos: Importância de ser um modelo leitor, Estratégias para leitura conjunta, Sugestões de ações para inserção da leitura na rotina, Compartilhamento de leituras por parte da família, Autosseleção do livro e Dicas para o incentivo do compartilhamento de leituras (apontados pela literatura como profícuos^3,4^).

Em seguida, elas foram convidadas a responder ao “Questionário de verificação de atitudes diárias de incentivo da leitura no ambiente familiar” disponibilizado de forma virtual pela ferramenta *Google Forms*. Na apresentação do *folder* digital, cada tópico foi explanado e exemplificado.

Por fim, foram apresentados quatro *sites* com livros virtuais gratuitos a fim de que elas escolhessem um livro e realizassem a leitura da história com seu respectivo filho, aplicando as estratégias discutidas no encontro.

### Resultados da etapa 2

A escolha, realizada pelos pacientes, de outro participante para interagir em etapas futuras foi realizada a partir da observação das fotografias e da leitura das frases de apresentação previamente elaboradas e postadas na Etapa 1. No caso de P1 e P4, a leitura da frase foi realizada pela Mediadora.

A leitura dos livros teve a duração de uma sessão de 30 minutos. P2, P3, P5 e P6 leram seus livros oralmente para a Mediadora. P1, optou por um livro com texto escrito. Dessa forma, o livro foi lido pela Mediadora com auxílio do paciente. Por fim, P4 optou por outro livro sem texto e a narração foi realizada por ele com pontuais intervenções da Mediadora.

O encontro do Grupo de mães teve a duração de 30 minutos e contou com a presença e envolvimento de todas as mães que se apresentaram e falaram sobre as dificuldades de seus filhos e sobre o que esperavam do encontro. Apontaram a satisfação em fazer parte do procedimento e o desejo de saber como fazer para favorecer o hábito leitor dos filhos. Após a explicação do folder digital, dúvidas foram colocadas e todas relataram o que já realizavam e o que gostariam de praticar sobre os tópicos abordados.

O “Questionário de verificação de atitudes diárias de incentivo da leitura no ambiente familiar” era composto por três perguntas e foi respondido por M1, M2, M3 e M4. A primeira pergunta era: “Com que frequência você costuma compartilhar o material que você leu com seu filho?”. M1 e M4 responderam “sempre” e M2 e M3 “raramente”. A segunda era “Seu filho/filha compartilha leituras que ele leu ou aprendeu?”, na qual foram obtidas as respostas “sim” pelas mães M2 e M4, e “às vezes” por M1 e M3. Por fim, a terceira questionava “Quais comportamentos você costuma ter diante das informações de leitura que seu filho te traz?” e disponibilizava cinco opções que poderiam ser assinaladas pelo entrevistado, sendo elas: paro o que estou fazendo para escutá-lo; escuto enquanto realizo outra atividade; complemento com mais informações; faço perguntas de entendimento; e faço elogios. Nesta pergunta, podia haver mais de uma resposta. M2 para o que está fazendo para escutar o filho; M1, M3 e M4 escutam seus filhos enquanto realizam outra atividade; M4 complementa com mais informações e faz perguntas de entendimento; M3 e M4 fazem elogios aos filhos.

Etapa 3: Na etapa três, cada paciente foi convidado a elaborar um resumo escrito sobre o livro lido na Etapa 2 e o enviar por meio da plataforma assíncrona ao paciente que o escolheu.

Nesta Etapa, o Grupo de mães participou de uma roda de conversa na qual foi estimulado a compartilhar experiências em relação ao livro lido e à utilização das estratégias discutidas no encontro anterior. Ao final, foi solicitado que respondessem novamente o “Questionário de verificação de atitudes diárias de incentivo da leitura no ambiente familiar”.

### Resultados da etapa 3

Grupo de pacientes: Foram necessárias duas sessões, de 30 minutos cada, para que os pacientes finalizassem os resumos e os enviassem aos pacientes que os escolheram.

Com P1 e P4, foram realizadas estratégias de reconto por meio de figuras, a fim de que eles fossem estimulados a narrar as histórias para a Mediadora e ela as transcrevesse em formato de resumo. Com os demais pacientes, a Mediadora realizou atividades orais de compreensão leitora, a fim de garantir que a história tivesse sido apreendida. Na mesma sessão, cada paciente escreveu em uma folha de papel o resumo de seu livro, fotografou-o e o enviou à Mediadora. Na sessão seguinte, a Mediadora valorizou a produção textual, trabalhou aspectos de aprimoramento e realizou adequações da ortografia para a norma culta, a partir de reflexões com a criança, quando necessário.

Grupo de mães: o intervalo de tempo entre o encontro dois e o três foi de sete dias. Neste encontro, participaram M2, M3 e M4 e a duração foi de 20 minutos. Apenas M4 realizou a leitura conjunta com a criança como foi proposto no encontro dois. Ela relatou que a própria criança escolheu o livro e que esse foi um fator que o motivou a ler. Além disso, acrescentou que o fato do livro ser virtual, aumentou o interesse. Dentre os seis tópicos abordados no encontro dois, a mãe buscou: estratégias de leitura conjunta, a autosseleção do livro e as dicas para o incentivo do compartilhamento de leituras.

Um aspecto relevante mencionado pela mãe foi que o intervalo de sete dias foi positivo, pois a motivou a aplicar em um curto período o que havia aprendido no encontro anterior. O maior desafio é conciliar afazeres domésticos com a educação e estimulação dos filhos.

M2 pontuou que, apesar de não ter realizado a atividade de leitura com o filho, aplicou nos sete dias passados, algumas estratégias abordadas no grupo de mães e incentivou a filha mais velha, que tem o hábito da leitura a conversar mais sobre leituras com o irmão (P2) e a lerem juntos. Afirmou que a família toda já possui o hábito de conversar sobre leitura, mas que agora passou a parar o que está fazendo para ouvir os relatos das leituras dos filhos.

M3 afirmou ser um modelo leitor, mas que nunca havia notado que ela não compartilhava com o filho suas leituras. Acrescentou ainda que estava motivada a praticar as orientações fornecidas no encontro dois, principalmente realizar a leitura conjunta. Ressaltou também a importância do Grupo de mães, pois muitas vezes ela achava que para fazer o filho ler era necessário apenas presenteá-lo com um livro. No entanto, naquele momento, ela compreendia as complexas dimensões envolvidas no hábito de ler e afirmou estar empenhada para praticar as estratégias de incentivo do compartilhamento de leituras.

Quanto à resposta ao “Questionário de verificação de atitudes diárias de incentivo da leitura no ambiente familiar”, apenas M3 e M4 o preencheram. Quando comparadas às respostas de M3 ao questionário, no encontro um (pré-intervenção) e no encontro três (pós-intervenção), foi observado que, na questão um, “Com que frequência você costuma compartilhar o material que você leu com seu filho?”, a resposta foi alterada de “raramente” para “sempre”. M4 manteve as respostas em ambos os momentos de aplicação do questionário, porém, na questão três, “Quais comportamentos você costuma ter diante as informações de leitura que seu filho te traz?”, um novo comportamento foi assinalado, sendo ele “Paro o que estou fazendo para escutá-lo”.

Etapa 4: Por fim, na etapa quatro, foi solicitado que cada participante lesse o resumo recebido, gravasse um vídeo de agradecimento e com comentários sobre a história e o enviasse para o colega pela plataforma assíncrona. Houve também a solicitação do reconto, para a mãe, do livro lido na etapa 2 e do preenchimento da “Escala de Comportamentos Leitores”. O reconto foi gravado e analisado, pelos pesquisadores que preencheram o “*Check-list -* Comportamentos dos pais frente ao relato da leitura do filho”. Portanto, nessa etapa, o Grupo de mães participou como ouvinte do relato do livro lido pelo filho na Etapa 2.

### Resultados da etapa 4

Esta etapa ocorreu em duas sessões de 30 minutos, na primeira foi realizada a leitura do resumo, gravação e envio do vídeo para o colega, e na segunda os pacientes assistiram ao vídeo enviado pelo colega, realizaram reconto do livro lido na Etapa 2 para as mães e preencheram a Escala de Comportamentos Leitores.

Para o conhecimento do resumo recebido, os pacientes não alfabetizados precisaram da ajuda da Mediadora para leitura do mesmo, e os alfabetizados, fizeram a leitura oral. Em relação aos vídeos que receberam, os pacientes afirmaram, de formas diferentes, que haviam ficado animados em conhecer os colegas pelos vídeos gravados e por ouvi-los sobre o livro que leram. O resultado obtido com a “Escala de Comportamentos Leitores”, pré e pós-aplicação do procedimento, pode ser visualizado na Tabela 2.

Os pacientes P2, P4, P5 e P6 contaram com a presença de suas respectivas mães na sessão, para reconto do segundo livro virtual lido. As interações das quatro díades que participaram: M3-P3, M4-P4, M5-P5, M5-P6, foram analisadas: houve a presença de contato ocular, de perguntas pertinentes e sorrisos por parte das mães nas quatro díades; incentivo à resposta e assentimento com a cabeça em três díades (M3-C3, M4-C4, M5-C6), em contraste aos registros da Etapa 1 no qual não foi observado contato ocular e foi apontado um registro de incentivo à resposta e assentir com a cabeça. Com relação a presença de outros comportamentos, houve demonstração de interesse e envolvimento (M3-C3), abraço e participação da mãe ao longo da leitura (M4-C4).

## DISCUSSÃO

A pronta concordância em participar das atividades de intervenção de leitura, pelas mães e pacientes participantes, o engajamento de todas as díades convidadas e a não desistência podem ser indicadores de que o procedimento, a condução dos temas e atividades foram de interesse e adequados ao propósito apresentado. Também sugerem que o comportamento leitor não é necessariamente aversivo, mas pressupõe procedimentos favorecedores ao engajamento, diferentemente de atividades relacionadas à comunicação oral que por si só é natural da comunicação humana.

Nesse sentido, é importante ressaltar que o procedimento não se mostrou aversivo nem mesmo para as crianças que apresentam problemas escolares voltados à alfabetização e que enfrentam dificuldades em outras dimensões além das impostas pelo distanciamento social^(15)^. Lidar com a situação de leitura para estes estudantes é enfrentar dificuldades de memória de trabalho fonológica, de acesso ao léxico na memória de longo prazo e com as habilidades metafonológicas, para citar as principais^(16,17)^.

Levando-se em consideração a situação de pandemia, o formato totalmente *online* da aplicação foi uma escolha positiva para que os pacientes não deixassem de ser acompanhadas, pois permitiu a estimulação em linguagem escrita e do hábito de leitura, de forma a dar continuidade às atividades de acordo com todas as suas avaliações e intervenções prévias.

Outra vantagem deste formato, que pode ter contribuído para a não evasão e o engajamento, foi a maior flexibilidade na oferta de horários e a redução de custos para as famílias, como por exemplo com deslocamento físico. Tais aspectos também foram observados em uma pesquisa brasileira^(2)^.

No entanto, para que a aplicação *online* fosse possível, houve a necessidade de se aplicar rigorosos critérios de inclusão, fazendo com que poucos pacientes fossem elegíveis. Uma importante discussão acerca do acesso à internet e às tecnologias é que ainda não é uma realidade de todos os pacientes socioeconomicamente menos favorecidos^(15)^, sendo uma limitação importante.

Outro fator que levou à exclusão de possíveis participantes foi a falta de disponibilidade de ter um adulto para acompanhar o participante durante as sessões remotas. Apesar de saber do importante impacto na exclusão de participantes em potencial no estudo, os pesquisadores mantiveram a decisão de controlar esta variável. A importância e o impacto do acompanhamento em atividades realizadas em casa têm sido discutidos e apontados como um fator determinante de sucesso no que diz respeito tanto ao contexto virtual^(18)^ como presencial e, quanto a crianças com e sem dificuldades escolares.

Os casos relatados dizem respeito a crianças e adolescentes com histórico de dificuldades no processo de aprendizagem da linguagem escrita. Este fato torna o acompanhamento ainda mais importante, já que alguns comandos por escrito não são possíveis e também porque a participação do adulto não apenas facilita o momento em si da intervenção, mas também possibilita que este adulto passe a agir de forma conectada com as propostas terapêuticas em outras situações cotidianas. Os dados deste estudo corroboram a ideia de que o engajamento pode ter sido favorecido pela participação ativa dos adultos.

Ao todo, a proposta era de que a criança experienciasse a leitura de três livros escolhidos por ela. O apontamento de que a possibilidade de autosseleção da literatura é um fator importante para garantir o engajamento^(8)^, foi observado ao longo das sessões e no relato de uma das mães participantes: “O livro que ele escolheu foi: Um Medo para Arrepiar, sobre coisas de monstro, então já incentivou o interesse dele”. Portanto, considera-se este um fator importante tanto para a prática clínica, quanto pedagógica e de estimulação em ambiente familiar.

Outro aspecto da plataforma *online* utilizada, que corrobora um estudo já apresentado^(8)^, é a utilização da modalidade assíncrona nas interações entre os usuários. As interações não-simultâneas permitiram maior tempo para leitura e cuidado com a escrita^(8)^. A assincronicidade foi positiva, pois permitiu que a Mediadora trabalhasse as particularidades da prática da leitura e da escrita de cada criança.

Além disso, a implementação de recursos audiovisuais nas interações em clubes do livro virtuais, a fim de auxiliar aqueles que possuem dificuldades com a linguagem escrita^(8)^ foi empregada e facilitou a participação de todos e a proximidade entre os participantes que ali experienciaram uma interação totalmente virtual.

Uma consideração relevante diz respeito ao papel desempenhado pela Mediadora. Ao analisar os resultados, é possível observar que a Mediadora conduziu a realização das atividades propostas e realizou adaptações de acordo com as necessidades e particularidades da leitura e escrita de cada um. A mesma situação se repetiu no contexto das rodas de conversas com as mães, em que foi necessária sensibilidade para conquistar a confiança das participantes e deixá-las à vontade para compartilharem suas experiências e dificuldades cotidianas, além de garantir a participação igualitária de todas. A Mediadora zelou para que se privilegiasse o compartilhamento das experiências em uma construção coletiva de aprendizagem a qual se mostrou necessária, inclusive no contexto das mães.

As análises das respostas das mães nas rodas de conversa evidenciam que elas compreendem que o acompanhamento familiar é essencial para o desenvolvimento do hábito leitor. No entanto, apresentavam poucos dos comportamentos apontados como facilitadores pela literatura, inicialmente, e após poucos encontros sobre o tema, passaram a incorporar ações importantes na interação com o filho. O presente estudo levou em consideração os apontamentos de estudos anteriores para programar os temas a serem levados aos acompanhantes^(4),(5)^, ao abordar os assuntos relacionados ao hábito de ler, alguns aspectos se tornam indispensáveis, sendo eles abordados no encontro dois da metodologia do grupo de mães: a comunicação explícita sobre o valor da leitura, a realização de escuta ativa e questionamentos, a criação de um ambiente favorável ao hábito de ler, a incorporação de práticas de leitura na rotina^(3)^, a importância da exposição a livros, desenvolvimento de assuntos escolares em casa, reserva de momentos para prática da leitura, prática da leitura em voz alta e o compartilhamento de leituras^(4)^. Desse modo, levanta-se a discussão sobre a importância não apenas de se ter um acompanhante para as atividades para casa, mas, também, da necessidade de que haja a ampliação do repertório dos familiares, para que esse acompanhamento seja mais bem aproveitado. Os dados desse estudo demonstram que quando há construção conjunta de repertório sobre atitudes cotidianas facilitadoras de aprendizagem, há um maior engajamento por parte do familiar e aumenta-se, assim, a chance de que a colaboração com as atividades dos filhos passe a ocorrer de forma menos aversiva para todos.

Quando comparada a escala de comportamentos leitores pré e pós intervenção, é possível notar uma mudança positiva na resposta de, pelo menos, duas situações de cinco crianças. Ou seja, após o procedimento, cinco delas passaram a responder positivamente a comportamentos que as tornam mais propensas a possuírem comportamentos leitores. Faz-se aqui uma ressalva, por entender-se que é possível que tal mudança tenha ocorrido, neste primeiro momento, apenas no âmbito do comportamento verbal, mas ainda não tenha ocorrido modificações nas ações da rotina. Nesse sentido, seria importante que acompanhamentos futuros fossem realizados com a díade.

Outra análise é a mudança de comportamento das quatro mães que participaram dos encontros um e quatro da metodologia. Tal mudança diz respeito à presença de comportamentos no encontro quatro não demonstrados no um, como: o contato ocular, a realização de perguntas, o incentivo a respostas, os sorrisos e os assentimentos com a cabeça. Das quatro mães, todas elas haviam participado do encontro dois e apenas duas do encontro três. Este fato gera a reflexão sobre a importância do encontro dois para a mudança de postura dessas mães e as consequências a curto e longo prazo no hábito de leitura dessas crianças.

Ainda sobre o grupo de mães, é importante ressaltar o relato da mudança nas atitudes diárias de incentivo à leitura apontadas pelas duas mães que responderam ao questionário. Uma mãe passou a compartilhar “sempre” suas leituras com o filho e a outra passou a “parar o que está fazendo para ouvi-lo”, já que são mudanças importantes para promover a formação do hábito de leitura.

Sugere-se que estudos semelhantes sejam realizados com um número amostral que permita análises estatísticas a fim de se mapear os efeitos do procedimento, a realização de estudos longitudinais com a exposição dos participantes a mais livros com o intuito de verificar a presença de mudanças nas escalas de comportamentos leitores após o tempo de estimulação e a aplicação do presente procedimento em um modelo presencial.

Por fim, uma última reflexão nos remete ao fato de que a situação pandêmica impôs inovações em diversas áreas e junto ao trabalho da Fonoaudiologia no que diz respeito à linguagem escrita não foi diferente. Apesar do objetivo deste estudo ter sido pontual: relatar as experiências da aplicação *online* de um procedimento de incentivo à leitura, em crianças com dificuldades escolares e seus respectivos responsáveis; seus resultados vem se somar a outros^(19)^ que reforçam a possibilidade da realização de intervenções profícuas por meio da telefonoaudiologia, perspectiva importante do ponto de vista econômico, social, educacional e que merece atenção especial a partir desse momento histórico pelo qual passa toda a área da saúde e da educação.

## COMENTÁRIOS FINAIS

As crianças e adolescentes com dificuldades escolares tiveram sua aprendizagem ainda mais comprometida durante o período de pandemia do COVID-19. Uma forma de diminuir os prejuízos com as dificuldades em linguagem escrita é por meio da leitura.

O presente relato mostrou os benefícios de um procedimento fonoaudiológico, aplicado no formato *online,* com enfoque no compartilhamento de leituras por meio de uma plataforma digital e no incentivo à leitura no ambiente familiar. Dentre os resultados obtidos estão: a mudança de comportamentos das crianças envolvidas para maior propensão a hábitos leitores, o relato de mudança de atitudes diárias das mães no ambiente familiar para o incentivo à leitura e a presença de comportamentos incentivadores por parte das mães frente ao compartilhamento de leituras de seus filhos.

As experiências relatadas mostraram-se promissoras. Para maior consolidação dos achados, sugerem-se novos estudos com número amostral que permita análises estatísticas, estudos longitudinais que meçam o impacto do procedimento a longo prazo e também a aplicação presencial ou híbrida do procedimento.
